# Unusual decay: Recombination loss leads to splicing errors in green algae

**DOI:** 10.1371/journal.pbio.3003851

**Published:** 2026-06-26

**Authors:** Anamaria Necsulea

**Affiliations:** Universite Claude Bernard Lyon 1, LBBE, UMR 5558, CNRS, VAS, Villeurbanne, France

## Abstract

Recombination suppression leads to genomic erosion through an accumulation of deleterious mutations. This Primer discusses a new study that reveals an outstanding increase in aberrant splicing in non-recombining genomic regions in green algae.

## Teaser

Recombination suppression leads to genomic erosion through an accumulation of deleterious mutations. A new study in *PLOS Biology* reveals an outstanding increase in aberrant splicing in non-recombining genomic regions in green algae.

How do DNA sequences evolve following the suppression of recombination? In eukaryotic organisms, recombination produces new allele combinations, increasing the efficiency of natural selection. The beneficial effects of recombination are best illustrated by the consequences of its loss: in non-recombining genomic regions (such as Y chromosomes in mammals or in fruit flies), slightly deleterious mutations are able to accumulate, leading to extensive genomic decay. The hallmarks of recombination suppression are gene order changes, repetitive element expansions, and gene losses [1]. In a new study published in *PLOS Biology* by Condon and colleagues [2], a previously unexplored genomic erosion mechanism is revealed in green algae: an extraordinary increase in aberrant splicing in non-recombining regions.

**Fig 1:**
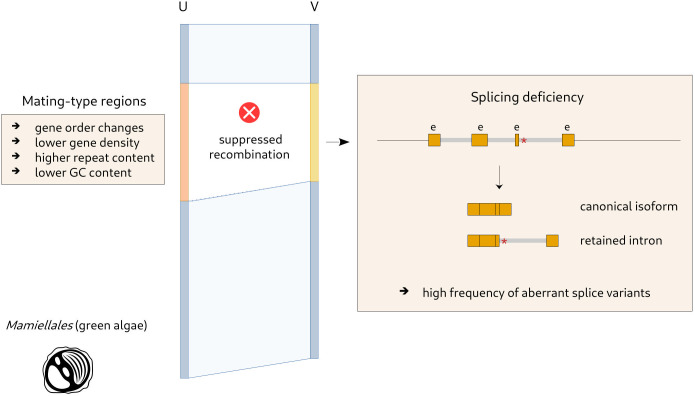
Splicing deficiency in non-recombining mating-type regions of green algae. In green algae from the *Mamiellales* order, the U and V sex chromosomes contain large non-recombining DNA segments, called mating-type (MT) regions. These regions contain sex-determining genes as well as numerous other genes. They share many characteristics with non-recombining sex chromosomes, such as the presence of genomic rearrangements, increased repetitive sequence content, and lower gene density. In their new article, Condon and colleagues reveal a previously unexplored feature of MT regions: a high frequency of aberrant splice variants. Genes localized in MT regions produce numerous transcripts that contain retained introns. These transcripts are likely non-functional. Condon and colleagues observed that the increase of aberrant splice variants is associated with the presence of degenerate splicing regulatory motifs (depicted by the * symbol). Moreover, MT regions appear to have low DNA methylation levels and more open chromatin, which can likewise interfere with splicing regulation. The species silhouette depicts *Ostreococcus tauri* and was downloaded from PhyloPic (https://www.phylopic.org/).

During their life cycle, the green algae in the *Mamiellales* order alternate between haploid and diploid stages. In these species, sex is genetically determined at the haploid stage: one sex possesses a U chromosome, while the other one carries a V chromosome [3]. The U and V chromosomes contain large non-recombining genomic segments called mating-type (MT) regions, which include the sex-determining gene as well as many other genes [4]. These regions share many characteristics with classical non-recombining sex chromosomes, including higher repetitive sequence content and lower gene density. Notably, MT regions have markedly lower GC content than their neighboring genomic regions or than autosomal chromosomes [4]. This can be explained by the absence of GC-biased gene conversion, a recombination-associated process that promotes the fixation of AT to GC mutations [5]. In their report, Condon and colleagues [2] reveal an additional intriguing characteristic of MT regions, related to their alternative splicing patterns (Fig 1).

Specifically, they found that genes localized in MT regions produce high numbers of transcripts that are aberrantly spliced, in particular transcripts where introns were not all properly excised—a phenomenon known as intron retention [6]. Strikingly, average intron retention frequencies were more than 30 times higher for genes localized within MT regions than for other genes. This pattern was remarkably conserved during evolution: despite having diverged more than 300 million years ago, all four analyzed *Mamiellales* species showed elevated intron retention frequencies in MT genes. Other types of alternative splicing events, such as the use of alternative splice sites, are likewise over-represented on MT regions. However, the abundance of intron-retaining isoforms is particularly noteworthy because these transcripts are unlikely to be translated into functional proteins, as they often induce reading frame shifts and contain premature stop codons. Thus, it appears that MT regions are undergoing functional genomic erosion: although they still contain hundreds of genes with intact sequences (in contrast with the highly degenerated Y chromosomes in mammals, which retain only a minuscule fraction of their ancestral gene content [1]), MT genes produce high frequencies of non-functional transcripts. How the production of these aberrant transcripts is tolerated by the minuscule green algae cells remains an open question. One likely hypothesis is that aberrant isoforms are detected and degraded by downstream control mechanisms, such as nonsense-mediated decay (NMD) [7]. This is a translation-coupled RNA quality control mechanism, which detects and degrades transcripts containing premature stop codons [7]. The core NMD machinery genes appear to be present and expressed in *Mamiellales* [2]. Future work is needed to determine if the aberrant splicing isoforms produced by MT regions are subject to degradation by NMD or if their production may be mitigated by additional post-transcriptional control mechanisms.

Condon and colleagues [2] suggest several intriguing hypotheses to explain the prevalence of aberrant splicing in MT regions. Interestingly, they observe that GC-rich splicing regulatory motifs are depleted in genes localized in MT regions. This could be explained by the absence of GC-biased gene conversion, which normally counteracts the universal AT-bias of mutational processes [5]. In addition, MT introns are shorter, a characteristic that is linked with reduced splicing efficiency in vertebrates [8]. Finally, they show that MT regions have reduced DNA methylation levels—at least in part due to a depletion in GC nucleotides—and thus more open chromatin, which may also interfere with splicing regulatory mechanisms [9]. Overall, these observations suggest that the observed splicing deficiency in MT regions may be the result of mutational processes that are not counteracted by biased gene conversion in the absence of recombination.

These intriguing observations highlight the different evolutionary trajectories of MT regions and ‘classical’ sex chromosomes. Differences between these two types of non-recombining genomic regions are expected for several reasons. In diploid organisms, sex chromosomes are expected to evolve differently from autosomes, not only due to the absence of recombination but also due to their lower effective population sizes. Moreover, recessive mutations are more visible to natural selection on sex chromosomes, which likewise shapes their evolution [1]. These factors cannot influence the evolution of MT regions in green algae, as in these species haploid stages are dominant and sexual reproduction is rare [4]. Moreover, MT regions are not expected to experience the dramatic gene losses observed on the highly degenerated Y chromosomes [1], as there are no gametolog copies to compensate for the loss. However, it is more difficult to explain why aberrant splice variants were so far not observed in Y chromosomes, as the loss of recombination should likewise prevent the action of GC-biased gene conversion and thus lead to the degeneration of GC-rich splicing regulatory motifs. The explanation may reside in the unusual chromatin state of MT regions, characterized by low DNA methylation levels. In vertebrates, differential DNA methylation between sex chromosomes and autosomes has been observed, and it may be involved in dosage compensation mechanisms [10]. However, low DNA methylation levels were so far not reported on degenerate Y chromosomes.

Condon and colleagues [2] hypothesize that splicing deficiency may appear at the initial stages of sex chromosome differentiation, prior to the establishment of more dramatic forms of genomic decay. Analyses of young sex chromosome systems, such as the neo-Y chromosomes observed in *Drosophila* species or the recently diverged X and Y chromosomes of *Silene latifolia* [1]*,* should provide a means to test this hypothesis. Future work is needed to determine whether the degradation of splicing regulation is a common feature in the evolution of non-recombining genomic regions or a unique trait of the *Mamiellales* genomes.

## Abbreviations

MT: mating-type
NMD: nonsense-mediated decay
